# Psychological well-being of adolescents in Pokhara, Nepal: A comparison between migrated and non-migrated parents

**DOI:** 10.1371/journal.pmen.0000102

**Published:** 2025-08-12

**Authors:** Anil Khanal, Bipin Thapa, Muskan Pudasainee, Bibash Rana, Poonam Paudel, Raju Prasad Sah, Prabin Shrestha, Nand Ram Gahatraj

**Affiliations:** 1 Pokhara University, School of Health and Allied Sciences, Pokhara, Kaski, Nepal; 2 Department of Child, Adolescent Health and Maternal Care, School of Public Health, Capital Medical University, Beijing, China; Assiut University, EGYPT

## Abstract

**Background:**

With the increasing trend of international migration in Nepal, there is a growing concern about the psychological well-being of left-behind adolescents. This study aimed to assess psychological well-being and identify factors associated with psychological well-being among adolescents of migrated and non-migrated parents in Pokhara Metropolitan, Nepal.

**Methods:**

A school-based cross-sectional study was conducted among 724 adolescents in the Pokhara Metropolitan. Adolescents were recruited through stratified random sampling. Socio-demographic, interpersonal, and migration-related factors were assessed. The pre-tested “Strengths and Difficulties Questionnaire” was used to measure the psychological well-being of the participants.

**Results:**

The parents’ migration status did not significantly affect the overall psychological well-being of adolescents in the study. However, females, relatively advantaged ethnic groups, non-Hindus, and those studying in private schools had significantly higher difficulty scores (p < 0.05). Higher levels of social support, especially from family, relatives, and teachers, and participation in extracurricular activities were associated with better psychological well-being (p < 0.05). Exposure to violence, feeling unsafe in the family, witnessing frightening behaviors due to substance use, and mistreatment by siblings were associated with poorer psychological well-being. More frequent and prolonged duration communication with migrated parents and discussion of life difficulties during communication were associated with better psychological outcomes in adolescents with migrated parents.

**Conclusions:**

The study found no significant difference in adolescents’ psychological well-being between migrated and non-migrated parents. However, various socio-demographic, interpersonal and migration-related factors play a role in shaping adolescents’ psychological well-being. The findings highlight the importance of social support, a safe family environment, and effective communication with migrated parents for the psychological well-being of left-behind adolescents in Nepal.

## Introduction

Adolescence is a critical developmental period in humans (ages 10–19 years according to World Health Organization (WHO) [1], though recent research extends this to 10–24 years, recognizing prolonged developmental processes) [2], characterized by significant physical, biological, neurological, psychological, and social changes. This transitional stage has specific health and development needs, requiring appropriate parental support [1]. However, increasing international migration around the globe potentially leaves many adolescents behind, raise significant concerns about their psychological well-being.

Globally, migration has surged in recent decades, and Nepal is no exception. As a major labor-exporting country, Nepal issued over 4.7 million labor permit between 2008 and 2022 [3]. According to the 2021 census, 23.4% of Nepalese households had at least one member living abroad, the majority of whom were male [4]. This migration trend has led to thousands of adolescents being left behind under the care of remaining parents or extended family members. Such arrangements may disrupt emotional support systems critical to healthy adolescent development.

In Nepal, mental health issues among adolescents are a significant public health concern. The 2020 National Mental Health Survey revealed that 5.2% of adolescents (aged 13–17 years) experience mental health disorders during their lifetime, with neurotic and stress-related disorders being the most prevalent (2.8%). Regarding suicidality, 3.9% reported current suicidal thoughts, and 0.7% reported lifetime suicidal attempts [5]. While these numbers appear lower, the 2010/11 Nepal Adolescents and Youth Survey (NAYS) used a broader definition of psychosocial problems and reported that 14% of adolescents experienced at least one psychosocial problem in the previous 12 months, including anxiety and restlessness (73.1%), feeling “fed up with life” (39.2%), negative thoughts and loss of self-confidence (38%), feelings of hopelessness (32.1%), and suicidal ideation (12%) [6]. Further emphasizing the severity of the situation, a retrospective study showed a threefold increase in the annual incidence of completed suicide among adolescents, from 5.1 per 100,000 in 2005 to 15.7 per 100,000 in 2009 [7].

Parents often migrate out of economic necessity to support their families through remittances, as evidenced by remittances comprising 31% of Nepal’s GDP in 2016. However, this economic survival strategy comes at a potential psychological cost to left-behind adolescents [8]. Bowlby’s attachment theory suggests that prolonged separation from parents can lead to psychological distress in children, manifesting as anxiety, anger, and depression [9]. Research indicates that left-behind adolescents may experience increased psychological and emotional stress, feelings of loneliness, and low self-esteem [6].

Despite the magnitude of parental migration and its potential impact on adolescent mental health, there is limited research specifically examining the psychological well-being of left-behind adolescents in Nepal. Most existing studies on left-behind children’s psychological well-being have been conducted in other countries, particularly China, and have focused primarily on internal migration [10]. The few studies in Nepal have primarily focused on comprehensive psychological well-being assessment rather than general health outcomes [11,12].

This study aims to assess the psychological well-being and factors associated with psychological well-being among adolescents of migrated and non-migrated parents in Pokhara, Nepal. Understanding these relationships is crucial for developing targeted interventions and support systems for this vulnerable population. The findings will provide evidence-based insights to inform policies and programs aimed at supporting the mental health needs of left-behind adolescents in Nepal.

## Methods

### Study design and settings

This school-based, cross-sectional study was conducted in Pokhara Metropolitan, Kaski district, Gandaki Province, Nepal. The Metropolitan area spans 464 square kilometers and has 33 wards, characterized by a heterogeneous and fluctuating population, massive migration, and segmentation of traditional social organization. In 2021,approximately 2,86,593 individuals (2,39,788 males and 46,805 females) migrated for foreign employment and were absent at home for at least 6 months in Gandaki Province of Nepal [4]. Specifically, in Pokhara metropolitan, at least one of the family members was absent due to foreign employment in 28.6 percent of the households during the same period of time [13].

### Study population

The study population consisted of adolescents (aged 12–17 years) studying in grades 8, 9, and 10 in selected secondary schools in Pokhara Metropolitan. Participants who were willing to consent and were currently studying in selected secondary schools were included in the study, while those unable to participate due to physical or psychological reasons were excluded. Written informed consent forms were provided to potential participants one day before the survey date. Participation required both written informed assent from adolescents and consent from their parents/guardians. Only students who brought parental consent and personal assent were included in the study. For adolescents with migrated parents, consent was obtained from their primary caregivers.

### Sample size and sampling technique

The sample size was calculated via OpenEpi software [14] based on the findings from a similar study in western Nepal [12]. The calculation assumed a power of 80%, a significance level of 5%, and a non-response rate of 10% (a 10% non-response rate was considered based on the findings from the tools’ pretesting, where potential non-responses were identified. We compared demographic characteristics of responders and non-responders where possible. The 10% non-response rate was primarily due to absence on survey days rather than refusal to participate, suggesting minimal systematic bias) [13]. The total sample size was 724 (362 per group). Stratified random sampling was used to select participants.

We obtained a list of secondary schools along with the number of students in each school from the District Education Office. A total of 10 schools were randomly selected from a list of 240 schools (100 public schools and 140 private schools) in Pokhara Metropolitan. To ensure equal representation, 5 public schools and 5 private schools were chosen. This approach allowed for a balanced consideration of both the public and private educational sectors, addressing potential differences that may influence adolescent well-being.

Within the selected schools, students in grades 8, 9, and 10 were stratified based on parental migration status, which was determined through school records and initial screening. Parental migration status was selected as the stratifying factor due to its known impact on socio-economic conditions, emotional support, and access to educational resources—factors that are closely related to adolescents’ psychological well-being [12,15,16]. Proportional sampling was then applied within each stratum, ensuring that the number of students selected from each group (migrated vs. non-migrated) reflected their actual representation in the total student population across grades 8, 9, and 10 within each school.

### Data collection and measures

Trained research assistants with backgrounds in public health collected the study dataset. They received two days of training on the study protocol, ethical considerations, and data collection tools. Data collection was scheduled during regular school hours in coordination with the school administration to minimize non-response. Students who met exclusion criteria based on physical or psychological conditions were identified through consultation with school authorities and excluded from the sampling frame. A structured questionnaire was used for data collection. The questionnaire included items to collect data on independent variables (parental migration status), dependent variables (psychological well-being of adolescents), and other covariates.

### Parental migration status

The primary independent variable in this study was parental migration status. Adolescents were asked if their father and/or mother had lived abroad for at least six months, including up to the data collection period. The responses were categorized as none of the parents living abroad, only the father living abroad, only the mother living abroad, or both parents living abroad. We acknowledge that our definition of ‘living abroad for 6 months’ may include heterogeneous migration patterns (seasonal vs. permanent). This limitation will be addressed in future research examining migration pattern subgroups.

### Psychological well-being of adolescents

The psychological well-being of adolescents was the dependent variable, and it was assessed using the self-reported Strengths and Difficulties Questionnaire (SDQ), a 25-item tool measuring emotional symptoms, conduct problems, hyperactivity/inattention, peer problems, and pro-social behavior. We analyzed the SDQ data using two complementary approaches. First, we used the established cutoff scores to categorize participants into normal (0–15), borderline (16–19), and high difficulties (20–40) groups for prevalence estimation. Second, we analyzed the total difficulties score (0–40) as a continuous variable to assess associations with various factors with greater statistical sensitivity, with higher scores indicating poorer psychological well-being. [17,18]. The SDQ has been validated in Nepal, and has shown acceptable reliability (Cronbach’s alpha = 0.69) [12]. The Nepali version has demonstrated good construct validity and cultural appropriateness for assessing adolescent psychological well-being in the Nepali context [19].

### Socio-demographic characteristics

Socio-demographic variables include age, gender, ethnicity, religion, parents’ educational status, economic status, and school type.

### Social support

Social support was measured by a scale of six items adapted from the Multidimensional Scale of Perceived Social Support (MSPSS). The adaptation process involved selecting essential items relevant to the Nepali adolescent context while maintaining the core constructs of the original MSPSS. The questions asked were as follows: whether or not there is someone who the child could turn to help (1) for difficulties in studying, (2) for personal problems, (3) if the child was teased or bullied, (4) if they were feeling sad or depressed, (5) for guidance in dealing with issues when things go wrong, and (6) to share happiness with. Children who scored 6, i.e., answered “yes” to all six items, were categorized as having high social support. Children who scored 3–5 were classified as medium social support, and those who scored 0–2 points were classified as low social support [16].

### International wealth index

Household wealth was measured through the International Wealth Index (IWI), a standardized asset-based wealth index. IWI value ranges from 0 to 100, and percentiles are used to categorize the ranges. The median values in each cutoff point were used for the category. Scoring: very low (≤63), low (64 - 76.99), middle (77 - 85.99), high (86 – 95.99), very high (96 – 100).

### Ethical considerations

Ethical approval for the study was obtained from the Institutional Review Committee, Pokhara University (Ref. No. 195/2080/81). Permission was taken from each school administration where the study was to be conducted, and written informed assent from participants and consent from parents/guardians were also obtained. Confidentiality and anonymity of the participants was ensured. The nature, purpose, and potential risks were clearly communicated, ensuring that the participants understood their right to withdraw at any time without facing negative consequences.

### Statistical analysis

The collected data were organized, coded, entered into Epi data, and imported into SPSS for analysis. Descriptive statistics were used to summarize the data. For categorical analysis, we classified participants into normal, borderline, and high difficulties groups based on established SDQ cutoff scores to determine prevalence rates. For subsequent analyses of associations, we used the continuous total difficulties score to maximize statistical power and sensitivity.

For bivariate analysis, the Mann-Whitney U test was used to compare mean total difficulties scores between two groups, and the Kruskal-Wallis test was used to compare more than two groups, as the outcome variables were not normally distributed. Chi-square tests were used only to compare the proportions of categorical variables.

In all multivariate analysis, ordinal logistic regression was used to model psychological well-being as a three-level outcome: high difficulties, borderline, and normal. The regression coefficients (β) represent the change in log-odds of being in the normal category versus higher difficulty categories for each unit change in the predictor variable. A positive coefficient indicates a higher chance of being in the normal category, reflecting better psychological well-being. Conversely, negative coefficient indicates a lower chance of being in the normal category, representing poorer psychological well-being (i.e., higher difficulties). Before proceeding with the regression, the assumption of proportional odds was tested using the Test of Parallel Lines and confirmed to be met, ensuring that the relationship between predictor variables and the outcome was consistent across all category thresholds. Variables showing significant associations (p < 0.05) in bivariate analysis were included in the multivariate model, with multicollinearity assessed using Variance Inflation Factor (VIF ≥ 2 considered acceptable) [20,21].A two-sided p-value less than 0.05 was considered statistically significant, with models’ goodness of fit assessed using Pearson and Deviance chi-square tests and explanatory power evaluated using Nagelkerke’s pseudo-R².

## Results

### Socio-demographic characteristics of adolescents

The study included 724 adolescents, with a mean age of 14.33 years (SD 0.969). Among them, 58.70% were early adolescents (12–14 years) and 41.30% were late adolescents (15–17 years). Both male and female participants were similar (females - 50.10%). The study population reflected Nepal’s diverse ethnic composition: Janajati (35.60%), representing indigenous ethnic groups with distinct cultural practices, followed by Brahmin (31.50%) and Chhetri (18.60%), who are traditionally considered higher caste groups with significant social influence; Dalit (12.40%), a historically marginalized group; and smaller proportions of Madhesi (1.10%) and Muslim (0.70%). Religious affiliation predominantly comprised Hindu (83.30%), followed by Buddhism (12.20%), Christianity (3.70%), and Islam (0.80%), mirroring the broader religious landscape of Nepal, which remains predominantly Hindu while maintaining religious diversity. The socio-economic status of the participants showed a balanced distribution across strata: high (27.10%), followed by very low (21%), middle (20.90%), low (19.30%), and very high (11.70%).Most participants (62.00%) were from nuclear families, while 33.00% belonged to joint families, and 5.00% lived in extended family arrangements, reflecting both traditional and evolving family structures in contemporary Nepali society. Over half of the participants (53.70%) attended private schools. Detailed descriptive statistics are provided in S1 Table under heading supporting information.

### Status of psychological well-being of adolescents

The psychological well-being of adolescents was first categorized into three groups based on SDQ cutoffs: normal, borderline, and high difficulties as shown in Table 1. The majority of adolescents (71.40%) fell into the normal category, with 68.8% among those with migrated parents and 74% among those with non-migrated parents. The borderline category accounted for slightly less than a fifth (17.10%) overall, 18.80% for migrated parents, and 15.50% for non-migrated parents. The high difficulties category represented slightly more than one in ten (11.50%) overall, 12.40% for migrated parents, and 10.50% for non-migrated parents. The data suggest that adolescents with migrated parents have a slightly higher proportion of borderline and high difficulties compared to those with non-migrated parents.

**Table 1:** Status of psychological well-being of adolescent.

Variables	Overall		Adolescents of Migrated Parents		Adolescents of Non-Migrated Parents	
	n	%	n	%	n	%
**Psychological wellbeing**						
Normal	517	71.40	249	68.80	268	74
Borderline	124	17.10	68	18.80	56	15.50
High Difficulties	83	11.50	45	12.40	38	10.50

### Factors associated with the psychological well-being of adolescents

For a more sensitive analysis of factors associated with psychological well-being, we utilized the continuous total difficulties score approach. As shown in Table 2, the bivariate analysis revealed no significant difference in total difficulty scores between adolescents with migrated and non-migrated parents. However, higher difficulty scores were observed among females (13 vs 11, p = 0.001), indicating poorer psychological well-being in female adolescents. Similarly, Janajati ethnic groups showed poorer psychological well-being than other groups (13 vs. 11–12, p = 0.003), as did non-Hindus compared to Hindus (13 vs. 12, p = 0.007), and those studying in private schools compared to government schools (12 vs. 11.5, p = 0.013).

**Table 2:** Factors associated with the psychological well-being of adolescents.

Characteristics		Adolescents of Migrated Parents				Adolescents of Non-Migrated Parents			Overall
		n		Median (Q_1_~Q_3_)	p-value	n	Median (Q_1_~Q_3_)	p-value	p-value
**Age**									
Early adolescents (12-14)		206		12(7~16)	0.15	219	11(8~16)	0.25	0.07^a^
Late adolescents (15-17)		156		13(9~16)		143	13(9~15)		
**Sex**									
Female		184		13(8.25~17)	0.07	179	13(9~17)	0.002*	**0.001** ^a*^
Male		178		11(8~16)		183	11(8~15)		
**Ethnicity**									
Brahmin/ Chhetri		155		10(7~16)	0.004*	208	12(8~15)	0.21	**0.003** ^b*^
Janajati		139		13(9~18)		119	12(9~16)		
Others		68		12.5(8.25~16)		35	10(8~16)		
**Religion**									
Hindu		293		12(8~16)	0.035*	310	12(8~15.25)	0.10	**0.007** ^a*^
Non-Hindu		69		13(10~18)		52	13(9~17.75)		
**Maximum education level of either parent**									
Can read and write only		12		11(7.50~14.50)		25	11.50(7~16)		0.52^b^
Basic school		71		11(7.50~14)		49	14(9~16)		
Secondary school		209		12(8~17)		167	12(9~15)		
Bachelor’s and above		70		14(8~18)		121	12(9~16)		
**Socio – economic status**									
Very low		72		12(8.25~15)	0.45	80	11(8~15)	0.51	0.41^b^
Low		74		11.50(7~17)		66	13(9~17.25)		
Middle		75		11(8~16)		76	12(8~15)		
High		117		12(8~17)		79	12(9~16)		
Very high		24		15.50(9~19.50)		61	12(9~15.50)		
**Type of family**									
Nuclear family		229		11(8~16)	0.12	220	13(9~16)	0.08	0.48^b^
Joint family		116		13(8~17)		123	11(8~14)		
Extended family		17		16(8.50~20.50)		19	12(7~16)		
**Type of schools**									
Private school		197		12(8~17)	0.13	192	12 (9~16)	0.049*	**0.013** ^a*^
Government School		165		12(7~16)		170	11.5(7.75~15)		
**Social Support and School performance related factors associated with the psychological well-being of adolescents**									
**Social Support**									
High		173		11(7~16)	0.037*	192	11(8~15)	0.008*	**0.001** ^b*^
Medium		163		13(9~16)		145	12(9~16)		
Low		26		13.50(8~18.25)		25	16(10.50~21.50)		
**Social Support From (Multiple Choice)**									
Family	Yes	329		12(8~16)	0.018*	328	12(8~15)	0.003*	**0.001** ^a*^
	No	33		14(10~18.50)		34	15(10~18.50)		
Friends	Yes	237		12(8~17)	0.84	241	12(8~16)	0.84	0.99^a^
	No	125		12(8~16)		121	12(9~15)		
Relatives	Yes	103		11(7~15)	0.006*	83	11(6~14)	0.002*	**0.001** ^a*^
	No	259		13(9~17)		279	12(9~16)		
Teacher	Yes	92		9(6~14)	0.001*	86	10(6~16)	0.002*	**0.001** ^a*^
	No	270		13(9~17)		276	12(9~16)		
**Participate in Extracurricular Activities**									
Yes		283		12(8~16)	0.10	290	12(8~16)	0.05	**0.009** ^a*^
No		79		14(10~17)		72	13(9.25~16)		
**Performance in Extracurricular Activity (n = 573)**									
Top		37		9(7~14)	0.15	32	11.50(8~15)	0.47	0.07^b^
Upper-middle		99		11(8~17)		112	11(7~15)		
Middle		131		12(9~17)		128	12(8~16)		
Lower-middle		11		11(9~17)		16	13.5(9~15.75)		
Low		5		10(5~18)		2	12		
**Academic Performance (n = 724)**									
Top		22		8.50(6~14.25)	0.031*	43	11(8~15)	0.010*	**0.001** ^b*^
Upper-middle		101		11(7~16)		132	11(8~14)		
Middle		203		13(9~16)		157	13(9~16.50)		
Lower-middle		31		12(8~18)		24	13.50(11~17.50)		
Low		5		17(6.5~21.50)		6	14(12.50~17)		
**Behavioral and Violence related factors associated with the psychological well-being of adolescents**									
**Tobacco product consumption in the last 30 days**									
Yes		5		15(12~17.50)	0.26	2	13	0.86	0.26^a^
No		357		12(8~16)		360	12(8~16)		
**Alcohol consumption in the last 30 days**									
Yes		8		16.5(11.25~23)	0.06	3	15	0.57	0.05^a^
No		354		12(8~16)		359	12(8~16)		
**Experienced any violence in the last six months**									
Yes		65		16(11~19)	0.001*	65	14(9.50~20)	0.003*	**0.001** ^a*^
No		297		11(7.50~16)		297	12(8~15)		
**Frequency of violence**									
Rarely		26	17(13.25~20.25)		0.41	15	12(8~15)	0.28	0.43^b^
Sometimes		35		16(11~18)		35	14(9~20)		
Frequently		4		16.50(9.75~24)		15	16(11~23)		
**Feel safe in your family**									
Yes		358		12(8~16)	0.29	353	12(8~16)	0.014*	**0.011** ^a*^
No		4		16(8.75~25.50)		9	16(13.50~20)		
**Anyone in home frighten you by using alcohol/drugs**									
Yes		15		14(8~18)	0.23	25	14(10~19)	0.028*	**0.015** ^a*^
No		347		12(8~16)		337	12(8~16)		
**Family members shouting in a frightening way**									
Yes		67		16(12~19)	0.001*	82	16(12~20)	0.001*	**0.001** ^a*^
No		295		11(7~16)		280	11(8~14)		
**Family members physically hurt each other**									
Yes		42		18(13.50~21)	0.001*	49	15(13~19.50)	0.001*	**0.001** ^a*^
No		320		12(8~16)		313	11(8~15)		
**Mistreated or bullied by siblings at home**									
Yes		39		16(12~18)	0.001*	42	14.5 (10.75~18)	0.002*	**0.001** ^a*^
No		323		12(8~16)		320	12(8~15)		
**Adolescent living arrangements related factors associated with the psychological well-being of adolescents**									
**Currently living with**									
Parents		276		12(8~16)	0.57	326	12(8~16)	0.85	0.74^b^
Family members other than parents		62		13.50(8~18)		20	11 (7.25~15.75)		
Relatives		12		10(8.25~17.75)		7	11(6~14)		
School hostels		12		10.50(7~17.75)		9	11(8~15.50)		
**Rate your current living conditions (physical aspects)**									
Very good		204		11(7~15)	0.001*	188	11(8~14)	0.001*	**0.001** ^b*^
Satisfactory		147		14(9~18)		160	13(9~16)		
Poor		8		14(7.50~19.25)		7	15(9~18)		
Very poor		3		23		7	16(12~20)		
**Relationship with primary caretaker**									
Very good		305		12(8~16)	0.004*	310	12(8~15)	0.07	**0.001** ^b*^
Satisfactory		54		15(9.75~18)		50	13.50(9~17.25)		
Poor		3		19		2	15		
**Primary caretaker is caring and supportive to you**									
Yes		358		12(8~16)	0.59	355	12(8~16)	0.07	0.09^a^
No		4		14(8.50~18.75)		7	15(11~20)		
**Do you have your brothers or sisters living with you**									
Yes		270		12(8~16)	0.62	267	12(8~16)	0.95	0.64^b^
No		27		12(9~14)		38	12(7.50~16)		
Don’t have brothers or sisters		65		13(8.50~17)		57	12(8.50~15)		
**If yes, do you think he/she is caring and supportive to you (n=537)**									
Yes		255		12(7~16)	0.007*	259	12(8~16)	0.004*	**0.001** ^a*^
No		15		16(11~20)		8	17.50 (15.25~20)		
**Number of siblings do you have (n=602)**									
1		195		12(8~16)	0.60	172	12(9~15.75)	0.62	0.29^b^
2		78		12.50 (8.75~16.25)		92	12(8.25~17)		
3		17		11(6.50~18.50)		24	12.5(7.25~16)		
4		4		10.50(5.50~11)		14	9(7.75~15.25)		
5		3		8		3	9		

^a^Mann–Whitney U test; ^b^ Kruskal–Wallis test; ^*^ p < 0.05 is significant.

Higher social support, especially from family (median 12 vs. 14–15, p = 0.001), relatives (median 11 vs. 12–13, p = 0.001), and teachers (median 9–10 vs. 12–13, p = 0.001), and participation in extracurricular activities (p = 0.009) were associated with better psychological well-being. Exposure to violence in the last six months (p = 0.001), feeling unsafe in the family (p = 0.011), witnessing frightening behaviors due to substance use (p = 0.015), witnessing family members shouting and screaming (p = 0.001) or physically hurting each other (p = 0.001), and mistreatment by siblings (p = 0.001) were associated with poorer psychological well-being. Living conditions perceived as less than very good and poorer relationships with primary caretakers were also linked to higher difficulties scores (p < 0.001). Having caring and supportive siblings was associated with better psychological outcomes (p = 0.001).

### Parental migration-related factors associated with the psychological well-being of adolescents

The study found significant associations between adolescents’ psychological well-being and the frequency of missing migrated parents, as well as the frequency and duration of communication with them, as detailed in Table 3. Adolescents who reported missing their migrated parents “very much” had lower median psychological well-being scores (12, IQR = 8) compared to those who missed them “a little bit” (13, IQR = 10) or “not at all” (16, IQR = 17) (p = 0.024). Daily communication with migrated parents was associated with higher median psychological well-being scores (11, IQR = 8) compared to less frequent communication: once a week (13, IQR = 9), twice a week (15, IQR = 7), and once a month (14, IQR = 13) (p = 0.001). Additionally, shorter communication durations (less than 20 minutes) were linked to higher median psychological well-being scores (13) compared to longer durations (11–13) (p = 0.001). Discussing life difficulties with migrated parents was also associated with better psychological well-being, reflected in a higher median psychological well-being score (13.5, IQR = 7) compared to those who did not discuss such topics (median = 11, IQR = 8) (p = 0.023).

**Table 3:** Parental migration-related factors associated with the psychological well-being of adolescents.

Variables		Frequency(n)	Median (Q_1_~Q_3_)	Test value	p-value
**Parental migration (n=724)**					
Yes		362	12(8~16)	64973	0.85^a^
No		362	12(8~16)		
**Migrated Parents (n=362)**					
Father only		294	12(8~16)	4.88	0.09^b^
Mother only		26	17(9.25~21)		
Both Parents		42	11.50(8~17)		
**Duration of parental migration (n=362)**					
Father (n=336)	< 5 years	153	11(8~16)	1.52	0.47
	5 - 10 years	69	12(7~15.50)		
	> 10 years	114	12(8.75~16.25)		
Mother (n=68)	< 5 years	43	14(8~18)	1.99	0.37
	5 - 10 years	19	10(5~18)		
	> 10 years	6	12(7.50~16.25)		
**Parental migrated country (n=362)**					
**Father**	Arabic countries	178	12(8~16.25)	0.69	0.95^b^
	Asian country	104	11(7.25~16)		
	Europe	32	12.50(8~16)		
	Australia	11	12(10~16)		
	USA	11	11(8~16)		
**Mother**	Asian country	26	13.50(9~17.25)	7.90	0.10^b^
	Arabic countries	20	14(8~20.25)		
	Europe	14	7.50(3~18)		
	Australia	5	13(10~20)		
	Others	3	21		
**Frequency of missing migrated parents (n=362)**					
Very much		293	12(8~16)	7.45	**0.024** ^b*^
A little bit		62	14(8.75~18.25)		
Not at all		7	16(9~26)		
**Frequency of communication with migrated parents (n=362)**					
Every day		248	11(7~15)	18.92	**0.001** ^a*^
Once in a week		45	13(9.50~18)		
Twice in a week		45	15(11.50~18)		
Once in a month		24	14(8.25~20.75)		
**Duration of communication (n=362)**					
< 20 min		158	13(8~18)	14.93	**0.001** ^b*^
20 min- 40 min		107	13(9~17)		
> 40 min		97	11(6~14)		
Time (Minutes) Mean ± SD			29.90±18.65		
**Mode of communication (n=362)**					
Video call		335	12(8~16)	4.51	0.11^b^
Phone call		21	14(10~18)		
Text message		6	9(6~11.50)		
**Topic of communication (Multiple Choice) (n=362)**					
Children's day-to-day activities	Yes	309	12(8~16)	7762.50	0.54^a^
	No	53	13(8~18)		
Academic performance	Yes	256	12(8~17)	13075.50	0.59^a^
	No	106	13(9~16)		
Life difficulties	Yes	102	13.5(9.75~17)	11221.50	**0.02** ^a*^
	No	260	11(8~16)		
Learning difficulties	Yes	104	13(9~17)	12507.50	0.31^a^
	No	258	12(8~16)		
Migrant parental life	Yes	72	12(8.25~16.75)	10114	0.68^a^
	No	290	12(8~16)		
Children's feelings	Yes	100	13(8.25~18)	11608	0.09^a^
	No	262	12(8~16)		
**Frequency of meeting migrated parents physically (n=362)**					
Every six months		29	11(9.50~16)	3.51	0.62^b^
Once a year		88	12(7.25~16)		
Every two year		125	12(7~17)		
Every three year		83	13(9~17)		
Not meet yet		29	12(9.50~16.50)		
Others		8	10.5(4.70~15.75)		
**Happy with your parental migration (n=362)**					
Yes		137	12(8~17)	14899.50	0.60^b^
No		225	12(8~16)		

^a^Mann–Whitney U test; ^b^ Kruskal–Wallis test; ^*^ p < 0.05 is significant.

### Multivariate analysis of factors associated with the psychological well-being of adolescents

In a multivariate analysis of the overall population, gender emerged as a significant factor, with males demonstrating better psychological well-being compared to females. School type also showed significance, as government school attendance was associated with better psychological well-being outcomes compared to private schools. Family environment factors demonstrated strong associations with adolescents’ psychological well-being (see Table 4). The absence of violence and family conflict (shouting/screaming) were strong protective factors, with freedom from family verbal conflict showing the strongest positive impact on psychological well-being. The presence of low social support significantly decreased psychological well-being compared to those with high social support. Other factors like extracurricular activities, academic performance, living conditions, and religious/ethnic background did not significantly influence psychological well-being.

**Table 4:** Multivariate analysis of factors associated with the psychological well-being of adolescents in the overall population.

Variables	Estimate(β)	Std. Error	95% CI	
			Lower bound	Upper bound
**Sex**				
Female (Ref)				
Male	0.52	0.18	0.17	0.88
**Types of school**				
Private school (Ref)				
Government school	0.39	0.19	0.02	0.75
**Participate in extracurricular activities**				
Yes (Ref)				
No	-0.08	0.21	-0.49	0.334
**Academic performance**				
Low (Ref)				
Top	1.24	0.72	-0.16	2.65
Upper middle	0.81	0.64	-0.45	2.06
Middle	0.43	0.63	-0.80	1.65
Lower middle	0.12	0.67	-1.20	1.44
**Experienced violence**				
Yes (ref)				
No	0.75	0.22	0.32	1.18
**Witnessing family members shouting and screaming**				
Yes (Ref)				
No	1.01	0.22	0.59	1.43
**Family members hurt each other**				
Yes (Ref)				
No	0.50	0.26	-0.02	1.01
**Being mistreated or bullied by siblings**				
Yes (Ref)				
No	0.30	0.27	-0.23	0.82
**Current living conditions- physical aspects**				
Very poor (Ref)				
Very good	0.73	0.71	-0.67	2.13
Satisfactory	0.26	0.71	-1.13	1.65
Poor	0.93	0.92	-0.87	2.72
**Social support**				
High (Ref)				
Low	-0.93	0.32	-1.56	-0.29
Medium	-0.14	0.19	-0.50	0.23
**Religion**				
Non-Hindu (Ref)				
Hindu	-0.16	0.26	-0.65	0.34
**Ethnicity**				
Others (Ref)				
Brahmin/ Chhetri	0.001	0.28	-0.55	0.55
Jananati	-0.32	0.29	-0.88	0.24

When examining adolescents with non-migrated parents specifically (see Table 5), being male had an even stronger positive association with psychological well-being compared to the overall population. The absence of family conflict remained the strongest protective factor, with an even greater positive impact than in the overall population. The protective effect of not experiencing violence was maintained. Compared with the overall population, low social support was more strongly negatively associated with psychological well-being, emphasizing the critical role of support systems for this group. Unlike the overall population, school type did not emerge as a significant factor, suggesting that different dynamics influence educational experience in this subgroup.

**Table 5:** Multivariate analysis of factors associated with the psychological well-being of adolescents in non-migrated parents.

Variables	Estimate(β)	Std. Error	95% CI	
			Lower bound	Upper bound
**Sex**				
Female (Ref)				
Male	0.83	0.27	0.29	1.36
**Type of school**				
Private school (Ref)				
Government school	0.30	0.27	-0.24	0.83
**Academic performance**				
low (Ref)				
Top	0.88	1.04	-1.16	2.93
Upper middle	0.48	0.94	-1.37	2.33
Middle	-0.05	0.92	-1.86	1.76
Lower middle	-0.19	1.02	-2.19	1.80
**Experienced violence**				
Yes (Ref)				
No	0.76	0.34	0.10	1.42
**Witnessing family members shouting and screaming**				
Yes (Ref)				
No	1.61	0.31	1	2.22
**Family members hurt each other**				
Yes (Ref)				
No	-0.01	0.40	-0.78	0.77
**Being mistreated or bullied by siblings**				
Yes (Ref)				
No	0.38	0.39	-0.39	1.16
**Current living conditions -physical aspects**				
Very poor (Ref)				
Very good	0.26	0.90	-1.51	2.02
Satisfactory	-0.40	0.90	-2.16	1.36
Poor	0.83	1.35	-1.81	3.48
**Social support**				
high (Ref)				
Low	-1.37	0.47	-2.28	-0.46
Medium	-0.21	0.29	-0.77	0.34

The analysis of adolescents with migrated parents revealed some differences in the factors associated with normal psychological well-being (see Table 6). The absence of violence maintained its protective effect with a similar magnitude. Unlike other groups, the absence of physical family conflict emerged as a significant factor, whereas verbal conflict (shouting/screaming) became a non-significant factor. Being from a Janajati ethnic group negatively impacted psychological well-being compared to others marginalized groups. Interestingly, missing migrated parents “very much” was associated with better psychological well-being compared to not missing them at all. Other factors, including academic performance, social support, and communication patterns with migrated parents, showed no significant associations.

**Table 6:** Multivariate analysis of factors associated with adolescents’ psychological well-being in migrated parents.

Variables	Estimate(β)	Std. Error	95% CI	
			Lower bound	Upper bound
**Academic performance**				
Low (Ref)				
Top	1.30	1.07	-0.80	3.40
Upper middle	0.87	0.91	-0.92	2.65
Middle	0.76	0.89	-0.98	2.50
Lower middle	0.33	0.94	-1.51	2.18
**Experienced violence**				
Yes (Ref)				
No	0.81	0.30	0.22	1.40
**Witnessing family members shouting and screaming**				
Yes (Ref)				
No	0.50	0.33	-0.15	1.14
**Family members hurt each other**				
Yes (Ref)				
No	0.85	0.39	0.09	1.61
**Being mistreated or bullied by siblings**				
Yes (Ref)				
No	0.46	0.39	-0.31	1.23
**Current living conditions (physical aspects)**				
Very Poor (Ref)				
Very good	2.20	1.54	-0.82	5.22
Satisfactory	1.89	1.53	-1.11	4.90
Poor	2.04	1.71	-1.31	5.40
**Social support**				
High (Ref)				
Low	-0.12	0.49	-1.08	0.85
Medium	-0.06	0.26	-0.56	0.45
**Religion**				
Non-Hindu (Ref)				
Hindu	-0.15	0.34	-0.82	0.51
**Ethnicity**				
Others (Ref)				
Brahmin/ Chhetri	-0.32	0.37	-1.05	0.41
Janajati	-0.81	0.37	-1.54	-0.08
**Missing migrated parents**				
Not at all (Ref)				
Very Much	1.59	0.78	0.07	3.12
A little bit	1.34	0.81	-0.25	2.93
**Frequency of communication with migrated parents**				
Once in a month (Ref)				
Every Day	0.60	0.47	-0.33	1.53
Once in a Week	0.05	0.53	-0.99	1.08
Twice in a Week	-0.10	0.54	-1.15	0.95
**Duration of communication**				
> 40 min (Ref)				
< 20 min	-0.61	0.35	-1.29	0.07
20–40 min	-0.63	0.37	-1.35	0.09

## Discussion

This study investigated adolescents’ psychological well-being in Pokhara, Nepal, comparing those with migrated and non-migrated parents. The primary finding was that parental migration status did not significantly affect the psychological well-being of adolescents. This null result suggests that parental migration, in and of itself, may not be a standalone risk factor for adolescent psychological distress in the Nepali context.

### Parental migration and psychological well-being

Our finding of no significant difference in psychological well-being between adolescents with migrant and non-migrated parents contrasts with several studies from other Asian contexts. For instance, a meta-analysis by Fellmeth et al. (2018) found that left-behind children in China experienced significantly higher rates of depression and anxiety compared to non-left-behind peers [8]. Similarly, studies in Sri Lanka have reported higher rates of emotional and behavioral problems among children of migrant workers [22]. However, our results align more closely with findings from Southeast Asia and Thailand, which found that children’s psychological well-being were not significantly impacted by parents’ current migration status when strong family support systems were in place [23]. This similarity might be attributed to shared cultural contexts where extended family networks often provide robust support systems. The resilience of Nepali adolescents may be attributed to the country’s robust joint family system and community support structures, which research has shown can provide crucial emotional and social support during parental absence [12,24].

### Vulnerability factors and gender dimensions

This study revealed important gender-based differences, with female adolescents showing greater psychological vulnerability than males, which aligns with the findings of multiple exiting literature. Prior research has shown that female LBC are more likely to experience depression and anxiety, particularly in contexts of parental absence and academic stress [8,15,25]. This gender disparity underscores the importance of considering sex as a factor in psychological well-being among left-behind adolescents.

Additionally, adolescents attending private schools reported poorer psychological well-being, which aligns with findings from Nepal indicating higher academic pressure in private institutions [12]. Although private schools are generally perceived to offer better education [26], they may also impose greater academic demands that negatively affect students’ mental health [27,28].

### Social support and protective factors

The results highlight the crucial role of social support in the psychological well-being of adolescents. Those with higher levels of social support, particularly from family, relatives, and teachers, exhibited better psychological well-being. These findings are consistent with previous research indicating that social support contributes significantly to adolescent well-being, particularly among left-behind children [10,15,24] These findings reinforce the importance of social support in enhancing adolescents’ psychological well-being, especially those left behind due to parental migration.

Engagement in extracurricular activities and better academic performance also correlated positively with psychological well-being. These findings are in line with earlier studies from Nepal and South Asia showing that structured activities and educational success enhance adolescents’ self-esteem and reduce psychological distress [29–31].

### Risk factors and environmental influences

Exposure to violence, unsafe family environments, substance abuse by adults, and sibling mistreatment emerged as significant risk factors for poor psychological well-being. These findings support previous evidence that such adverse experiences contribute to higher rates of mental health problems in adolescents [32,33]. A study from rural Nepal similarly reported that frequent parental scolding and poor peer relationships significantly increased the likelihood of depression [31]. The absence of parents, as highlighted in the UNICEF report, exacerbates these vulnerabilities by weakening family structure and supervision, thereby increasing risks of neglect, abuse, and exploitation [34].

### Communication patterns and parental relationships

For adolescents with migrated parents, our finding that communication patterns (frequency and duration) significantly influence psychological well-being is supported by various stuties. A cross-sectional study in China found that more frequent and prolonged communication with migrated parents could significantly decrease depressive symptoms [24,35]. Similarly, Su et al. explain that communication plays a significant role in coping with emotional and behavioral problems. Children who are in harmonious communication with their parents tend to be happier [36]. The protective effect of discussing life difficulties during these communications suggests that the quality of interaction may be as important as frequency, supporting findings from Benjamin, 2016 [24,37].

### Strengths and limitations

This study makes a significant contribution to the limited research on the psychological well-being of left-behind adolescents in Nepal. The use of a validated psychological assessment tool (SDQ) and the inclusion of a matched comparison group of adolescents with non-migrated parents strengthened the validity of the findings. The sample size was sufficiently large to detect meaningful differences, and the stratified sampling approach ensured the inclusion of representative participants across school types.

However, this study has several important limitations. First, the school-based sampling frame excludes out-of-school adolescents who may be more vulnerable, limiting generalizability to all adolescents. Second, the cross-sectional design prevents the establishment of causality between migration and psychological outcomes. Third, self-reported measures may be subject to social desirability bias, particularly when reporting sensitive issues like family conflict. Additionally, the study’s urban setting in Pokhara may not represent rural areas where migration impacts could differ due to varying support systems and socioeconomic conditions.

To address potential sampling bias, we employed a stratified random sampling approach, with schools as clusters and stratification to ensure balanced representation of both public and private institutions. Although we adjusted for the complex survey design by incorporating random effects for schools and accounting for stratification, the random effect variance was found to be negligible. Model comparisons using the AIC and BIC further indicated that the fixed-effects model provided a better fit to the data. This conclusion was reinforced by a likelihood ratio test, which showed no significant improvement when random effects were included. While our analyses indicated that random effects did not substantially improve model fit in this particular dataset, we recommend that future research continue to consider complex survey design, including stratification and clustering adjustments, to better account for the nested nature of school-based data. Finally, this study did not pre-register the primary research question, which may introduce bias in reporting. Future studies should consider pre-registration to enhance transparency.

### Future research priorities

Future research should explore psychological well-being in rural settings where migration impacts may differ, examine longitudinal effects of parental migration to establish causal relationships, investigate effective interventions for left-behind adolescents, and compare impacts of different migration patterns including seasonal work, temporary stationed employment (ships, construction), and permanent migration, while distinguishing between subgroups based on parent-child contact frequency and migration duration. Studies should also explore the role of technology in maintaining parent-child relationships and how cultural practices in Nepal might buffer against negative effects of parental migration.

### Conclusion and implication

Contrary to popular assumptions, our study found no significant psychological well-being difference between adolescents with migrated versus non-migrated parents. This suggests that other mediating factors such as social support, school environment, and family dynamics may play a more critical role.

The findings highlight the need for targeted interventions that strengthen existing support systems, address gender disparities in psychological vulnerability, facilitate quality communication between migrated parents and children, and promote psychologically healthy educational environments. By addressing these factors, stakeholders can better support the well-being of adolescents in Nepal’s increasingly migration-influenced society.

## Supporting information

S1 Data Data Set. (SAV)

S2 Data Questionnaire. Psychological Well-being of Adolescents in Pokhara: A Comparison between Migrated and Non-migrated Parents.
(DOCX)

S3 Data Strengths and Difficulties Questionnaire. (PDF)

S4 Data STROBE Checklist. STROBE Statement—Checklist of items that should be included in reports of *cross-sectional studies*.
(DOCX)

S1 Table Socio-demographic characteristics of adolescents. (DOCX)
