# Supplementary material for: Psychological well-being of adolescents in Pokhara, Nepal: A comparison between migrated and non-migrated parents
Source: PLOS Ment Health. 2025 Aug 12;2(8):e0000102. doi: 10.1371/journal.pmen.0000102 (PMC12798523; doi:10.1371/journal.pmen.0000102)
Supplement: S2 Data — Psychological Well-being of Adolescents in Pokhara: A Comparison between Migrated and Non-migrated Parents. (DOCX) [file pmen.0000102.s002.docx]

Research Title: Psychological Well-being of Adolescents in Pokhara: A Comparison between Migrated and Non-migrated Parents

Questionnaire (प्रश्नावली)

Participant ID (सहभागी आई. डी): ………………

Name of the School (विद्यालयको नाम): ..................................................... Class (कक्षl) .....

Please circle (O) the most appropriate answer for the following questions about you and your family. (कृपया तपाईं र तपाईंको परिवारको बारेमा निम्न प्रश्नहरूको लागि सबैभन्दा उपयुक्त उत्तर (O) सर्कल गर्नुहोस्)

| **A.** | **Socio-demographic Information (सामाजिक-जनसांख्यिकीय** विवरण**)** | | |
| --- | --- | --- | --- |
| **S.N.**  (क्र.सं) | **Questions**  **(प्रश्नहरू)** | **Answers**  **(उत्तरहरु)** | **Skip** |
| A1 | Date of birth (जन्ममिति)  or  If don’t know, what is your completed age (in years)? (यदि थाहा छैन भने तपाई कति वर्ष पूरा हुनुभयो?) |  |  |
| A2 | Sex (लिंग) | 1. Male (पुरुष) 2. Female (महिला) |  |
| A3 | Which ethnicity do you belong to? (तपाईं कुन जातिको हुनुहुन्छ?) | 1. Brahmin (ब्राह्मण) 2. Chhetri (क्षेत्री) 3. Dalit (दलित) 4. Janajati (जनजाती) 5. Madhesi (मधेशी) 6. Muslim (मुस्लिम ) 7. Others (अन्य खुलाउनुहोस्) ....................................... |  |
| A4 | Which religion do you follow?  ( तपाईं कुन धर्म मान्नुहुन्छ?) | 1. Hinduism (हिन्दू धर्म) 2. Buddhism (बौद्ध धर्म) 3. Islam (इस्लाम धर्म) 4. Christianity (ईशाई धर्म) 5. Others (अन्य खुलाउनुहोस्) ....................................... |  |
| A5 | What is the highest level of your father's education?  (तपाईंको बुबाको शिक्षाको उच्चतम स्तर के हो?) | 1. Illiterate (निरक्षर) 2. Can read and write only   (पढ्न र लेख्न मात्र सक्ने)   1. Pre-Primary (पूर्व-प्राथमिक) 2. Basic school (1-8) ( आधारभूत तह (१-८) 3. Secondary school(9-12)(माध्यमिक तह) 4. Bachelors and above (स्नातक र माथि) |  |
| A6 | What is the highest level of your mother's education?  (तपाईंको आमाको शिक्षाको उच्चतम स्तर के हो?) | 1. Illiterate (निरक्षर) 2. Can read and write only (पढ्न र लेख्न मात्र सक्ने) 3. Pre-Primary (पूर्व-प्राथमिक) 4. Basic school (1-8) ( आधारभूत तह ) 5. Secondary school(9-12)(माध्यमिक तह) 6. Bachelors and above (स्नातक र माथि) |  |
| A7 | What type of family do you live in?  (तपाईं कस्तो परिवारमा बस्नुहुन्छ?) | 1. Nuclear family (एकल परिवार) 2. Joint family (संयुक्त परिवार) 3. Extended family (ब्रिह्यत परिवार) |  |
| A8 | What type of schools do you read?  (तपाईं कस्तो प्रकारको स्कूल पढ्नुहुन्छ?) | 1. Government School (सरकारी विद्यालय) 2. Boarding school (बोर्डिङ स्कूल) |  |

| **B.** | **Household Information (पारिवारिक जानकरी)** | | |
| --- | --- | --- | --- |
| **S.N.**  (क्र.सं) | **Questions**  **(प्रश्नहरू)** | **Answers**  **(उत्तरहरु)** | **Skip** |
| B1 | Does your father currently live with you?  (के तपाईंको बुबा अहिले तपाईंसँगै बस्नुहुन्छ?) | 1. Yes (बस्नु हुन्छ) 2. No (बस्नु हुदैन) | Go to  **B3** |
| B2 | If not, why doesn't your father live with you?  (यदि बस्नु हुदैन भने, तपाईंको बुबा तपाईंसँग किन बस्नुहुन्न?) | 1. Living abroad (विदेशमा भएर) 2. Living in another place in Nepal (Job, business, etc.) (नेपालमा अर्कै ठाउँमा भएर (जागिर, व्यवसाय आदि) 3. Parents' divorce (माता-पिताको सम्बन्ध बिछेद भएर) 4. Father not alive (बुबा जीवित हुनुहुन्न) 5. Others (अन्य खुलाउनुहोस्) ………. | Go to **B4** |
| B3 | What is your father's current main occupation?  (तपाईंको बुबाको मुख्य पेशा अहिले के हो? | 1. Agriculture (कृषि) 2. Business (व्यापार) 3. Labour work (श्रम कार्य) 4. Service (Private/Government)   सेवा (निजी/सरकारी)   1. Others (अन्य खुलाउनुहोस्) ……….……………… |  |
| B4 | Does your mother currently live with you?  (के तपाईंको आमा अहिले तपाईंसँगै बस्नुहुन्छ? | 1. Yes (बस्नु हुन्छ) 2. No (बस्नु हुदैन) | Go to **B6** |
| B5 | If not, why doesn't your mother live with you?  (यदि बस्नु हुदैन भने, तपाईंको आमा तपाईंसँग किन बस्नुहुन्न?) | 1. Living abroad (विदेशमा भएर) 2. Living in another place in Nepal (Job, business, etc.) (नेपालमा अर्कै ठाउँमा भएर (जागिर, व्यवसाय आदि) 3. Parents' divorce (माता-पिताको सम्बन्ध बिछेद भएर) 4. Mother not alive (आमा जीवित हुनुहुन्न) 5. Others (अन्य खुलाउनुहोस्) ………. | Go to **B7** |
| B6 | What is your mother's current main occupation?  (तपाईंको आमाको मुख्य पेशा अहिले के हो?) | 1. Agriculture (कृषि) 2. Business (व्यापार) 3. Housewife (गृहिणी) 4. Labour work (श्रम कार्य) 5. Service (Private/Government)   सेवा (निजी/सरकारी)   1. Others (अन्य खुलाउनुहोस्)   ………………………………….. |  |
| B7 | Tentative monthly income of your family? (तपाईंको परिवारको मासिक अनुमानित आम्दनी कति छ?) | .................................................(NRS) |  |
| B8 | Which of these items does your family have? **(Multiple choice)**  तपाईंको घरमा यी मध्ये कुनकुन सामान छ? **(बहुउत्तर दिनुहोस)** | 1. Television (टेलिभिजन) 2. Air conditioner (एयर कंडीशनर) 3. Refrigerator (रेफ्रिजरेटर) 4. Two wheelers (Bike/ Scooter) {दुईपाङ्ग्रे सवारी (बाइक / स्कूटर)} 5. Four wheelers (Car, jeep) {चारपाङ्ग्रे सवारी (कार, जिप)} 6. Computer/ Laptop (कम्प्युटर/ ल्यापटप) 7. Electricity (बिजुली) |  |
| B9 | What kind of floor is your house? (तपाई को घर को भुई कस्तो प्रकार को छ) | 1. Kacchi (कच्ची) 2. Cemented (पक्कि) 3. Tile/marble (टाइल / मार्बल) |  |
| B10 | How many rooms are used for sleeping in your house? (तपाईंको घरमा सुत्ने कोठा कति वटा छन्?) | ............................ |  |
| B11 | **Did you get social support in the following situations?**  **(के तपाईंले निम्न परिस्थितिहरूमा सामाजिक समर्थन पाउनुहुन्छ?)** | |  |
| B11a | When you face difficulties in studying (अध्ययनमा कठिनाइहरू हुँदा) | 1. Yes (हुन्छ) 2. No (हुदैन) |  |
| B11b | When you get personal problems  (व्यक्तिगत समस्याहरूमा) | 1. Yes (हुन्छ) 2. No (हुदैन) |  |
| B11c | If the child was teased or bullied you (तपाईलाई कसैले जिस्काउदा वा धम्की दिदा) | 1. Yes (हुन्छ) 2. No (हुदैन) |  |
| B11d | When you feel sad or depressed  (तपाई दुःखी वा उदास महसुस गरेको बेला) | 1. Yes (हुन्छ) 2. No (हुदैन) |  |
| B11e | For guidance in dealing with issues when things go wrong  ( समस्या पर्दा समस्या समाधानका लागि) | 1. Yes (हुन्छ) 2. No (हुदैन) |  |
| B11f | To share happiness with  (खुशी साटासाट गर्न) | 1. Yes (हुन्छ) 2. No (हुदैन) |  |
| B12 | From whom did you get social support mostly? **(Multiple choice)** {सामाजिक सहयोग कसकसबाट पाउनुहुन्छ?} **(बहुउत्तर दिनुहोस)** | 1. Family (परिवार) 2. Friends (मित्र) 3. Relatives (नातेदार) 4. Teacher (शिक्षक/शिक्षिका) 5. Others (अन्य खुलाउनुहोस्)   ……….……………… |  |
| B13 | Do you participate in extracurricular activities organized by your school? (के तपाईं आफ्नो विद्यालयले आयोजना गर्ने अतिरिक्त क्रियाकलापहरूमा भाग लिनुहुन्छ?) | 1. Yes (लिन्छु) 2. No (लिदीन | Go to **B15** |
| B14 | What type of extracurricular activities do you participate? **(Multiple choice)**  (तपाईं कस्ता प्रकारका अतिरिक्त क्रियाकलापहरूमा भाग लिनुहुन्छ?)  **(बहुउत्तर दिनुहोस)** | 1. **Performing arts (कला प्रदर्शन)** 2. **Sports/athletics (खेल/एथलेटिक्स)** 3. **Publications (प्रकाशन)** 4. **Academic clubs (शैक्षिक कल्ब)** 5. Others (अन्य खुलाउनुहोस्)   ………………………….. |  |
| B14 | what is your performance in extracurricular activities?  (अतिरिक्त क्रियाकलापहरूमा तपाईंको प्रदर्शन कस्तो छ?) | 1. Top (उत्कृष्ठ) 2. Upper-middle (राम्रो) 3. Middle (मध्यम) 4. Lower-middle (निम्न-मध्यम) 5. Low (कमजोर) |  |
| B15 | what is your academic performance level in your class?  (कक्षामा तपाईंको शैक्षिक प्रदर्शन स्तर कस्तो छ?) | 1. Top (उत्कृष्ठ) 2. Upper-middle (राम्रो) 3. Middle (मध्यम) 4. Lower-middle (निम्न-मध्यम) 5. Low (कमजोर |  |

| **C.** | **Behavior and Violence related Questions (व्यवहार र हिंसासँग सम्बन्धित प्रश्नहरु)** | | |
| --- | --- | --- | --- |
| **S.N.क्र.स.** | **Questions**  **(प्रश्नहरू)** | **Answers**  (उत्तरहरु) | Skip |
| C1 | Did you consume tobacco product in the last 30 days? (के तपाईंले पछिल्लो ३० दिनमा सुर्तीजन्य पदार्थ सेवन गर्नुभएको छ ?) | 1. Yes (छ)  2. No (छैन) |  |
| C2 | Did you drink alcohol in the last 30 days? (के तपाईंले पछिल्लो ३० दिनमा रक्सी पिउनुभएको छ?) | 1. Yes (छ)  2. No (छैन) |  |
| C3 | Have you ever experienced any violence in the last six months? (के तपाईंले विगत ६ महिनामा कुनै किसीमको हिंसाको अनुभव गर्नुभएको छ?) | 1. Yes (छ)  2. No (छैन) | Go to C7 |
| C4 | If yes, what types of violence did you face? (यदि छ भने तपाईंले कस्ता कस्ता प्रकारका हिंसाको सामना गर्नुपर्यो?  (बहुउत्तर दिनुहोस) | 1. Physical violence (शारीरिक हिंसा - हिर्काउनु, मुक्का हान्नु वा लात हान्नु)  2. Sexual Violence (यौन हिंसा)  3. Cyberbullying (साइबर बुलिंग)  4. Boycott (बहिस्कार)  4. Discrimination (विभेद)  5. Trafficking and Exploitation (मानव बेचबिखन तथा ओसारपसार)  6. Others (अन्य खुलाउनुहोस्)  ………………………….. |  |
| C5 | From whom did you often experience violence? (तपाईंले कसबाट हिंसा अनुभव गर्नुभयो?)  (बहुउत्तर दिनुहोस) | 1. Family members (परिवारका सदस्य)  2. Friends (साथीहरु)  3. Relatives (नातेदार)  4.Teacher (शीक्षक/शीक्षिका)  5.Others (अन्य खुलाउनुहोस्)  ………………………….. |  |
| C6 | During the past six months, how often did you face any types of violence? (विगत छ महिनामा तपाईंले कति पटक कुनै प्रकारको हिंसाको सामना गर्नुपऱ्यो?) | 1. Rarely (विरलै)  2. Sometimes (कहिलेकाहीं)  3. Frequently (अक्सर)  4. Daily (दैनिक) |  |
| C7 | Do you feel safe in your family?  के तपाईं आफ्नो परिवारमा सुरक्षित महसुस गर्नुहुन्छ? | 1. Yes (गर्छु)  2. No (गरर्दिन) |  |
| C8 | Has anyone in your home behaved in a way that frightened you by using alcohol or drugs? (के तपाईको घरमा कसैले मदिरा वा लागूपदार्थ प्रयोग गरी तपाईलाई डराउने तरिकाले व्यवहार गरेको छ?) | 1. Yes (छ)  2. No (छैन) |  |
| C9 | Have you seen family members in your home shouting and screaming in a way that frightened you? (के तपाईंले आफ्नो घरका परिवारिक सदस्यहरूलाई डराएको र चिच्याएको देख्नुभएको छ?) | 1. Yes (छ)  2. No (छैन) |  |
| C10 | Have you seen family members in your home hurt each other physically (e.g. hitting, slapping, and kicking)? (के तपाईंले आफ्नो घरमा परिवारका सदस्यहरूले एकअर्कालाई शारीरिक रूपमा चोट पुर्‍याएको देख्नुभएको छ (जस्तै हिर्काउने, थप्पड हानेर, लात हानेको)? | 1. Yes (छ)  2. No (छैन) |  |
| C11 | Have you been mistreated or bullied by your brother(s) or sister(s) at home? (के तपाईलाई घरमा तपाईका दाजुभाई वा दिदिबहिनी बाट दुर्व्यवहार गरिएको छ?) | 1. Yes (छ)  2. No (छैन) |  |

| D | Parental international migration status (बुवाआमाको अन्तर्राष्ट्रिय बसाईसराईको अवस्था) | | | |
| --- | --- | --- | --- | --- |
| **S.N.**  (क्र.सं) | **Questions**  (प्रश्नहरू) | **Answers**  (उत्तरहरु) | | Skip |
| D1 | Does your parents live abroad? (के तपाईंका आमा /बाबु/आमाबाबु विदेशमा हुनुहुन्छ? | 1. Yes (हुनुहुन्छ) 2. No (हुनुहुदैन) | | Go to E1 |
| D2 | Migrated parent  (विदेशमा रहनुभएको अभिभावक) | 1. Father only (बुवा मात्र) 2. Mother-only (आमा मात्र) 3. Both parents (आमाबाबु दुवै) | |  |
| D3 | How long has he/she been living abroad? (उहाँहरु कहिलेदेखि विदेशमा हुनुहुन्छ?) | **Father(बुवा)**  .......Years………Month | Mother(आमा)  .......Years………Month | |
| D4 | Which country is your father/mother living in now? (तपाईंका आमा/बाबु अहिले कुन देशमा हुनुहुन्छ? | **Father(बुवा)**   1. Arabic countries (अरबी देशहरू) 2. Europe (युरोप) 3. Asian country (एसियाली देश) 4. Others (अन्य खुलाउनुहोस्) ................................. | Mother(आमा)   1. Arabic countries (अरबी देशहरू) 2. Europe (युरोप) 3. Asian country (एसियाली देश) 4. Others (अन्य खुलाउनुहोस्) ................................ | |
| D5 | How much do you miss your migrated parents? (विदेशमा हुनुहुने आमा / बाबुलाई कत्तिको याद गर्नुहुन्छ?) | 1. Very much (निकै धेरै) 2. A little bit (अलिअलि) 3. Not at all (अलिकति पनि होइन) | |  |
| D6 | How often do you talk to your migrated parents? (विदेशमा हुनुहुने आमाबाबुसित तपाईं कत्तिको कुरा गर्नुहुन्छ?) | 1. Every day (हरेक दिन) 2. Once in a week (हप्तामा एक पटक) 3. Twice in a week (हप्तामा दुई पटक) 4. Once in a month (महिनामा एक पटक) 5. Others (अन्य खुलाउनुहोस्) .................... | |  |
| D7 | Duration of communication  (विदेशमा हुनुहुने आमाबाबुसित तपाईंको कुरा हुदा कति समय कुरा गर्नुहुन्छ?) | ……………. minute (मिनेट) | |  |
| D8 | How do you usually talk to your migrated parents? (विदेशमा हुनुहुने आमाबाबुसित कुरा गर्दा तपाईं प्रायजसो कसरी कुरा गर्नुहुन्छ ?) | 1. Text Message (Message मार्फत) 2. Phone call (फोन कल मार्फत) 3. Video call (भिडियो कल मार्फत) | |  |
| D9 | Topic of communication  **(Multiple choice)**  (विदेशमा हुनुहुने आमाबाबुसित के विषय धेरै कुरा गर्नु हुन्छ?)  **(बहुउत्तर दिनुहोस)** | 1. Children's day-to-day activities (दैनिक क्रियाकलाप) 2. Academic performance (शैक्षिक गतिविधि) 3. Life difficulties (जीवनका कठिनाइहरू) 4. Learning difficulties (सिकाइ मा भएका कठिनाइहरू) 5. Migrant parental life (आप्रवासी अभिभावकको जीवन) 6. Children's feelings (बालबालिकाको भावना) | |  |
| D10 | How often do you meet physically to your migrated parents? (विदेशमा हुनुहुने आमाबाबसंग तपाईं भेट कत्तिको हुने गर्छ?) | 1. Every six monthly (वर्षमा दुई पटक) 2. Once a year (वर्षमा एक पटक) 3. Every two years (प्रत्येक दुई वर्षमा) 4. Every 3 years (प्रत्येक ३ वर्षमा) 5. Others (अन्य खुलाउनुहोस्) ....................................... | |  |
| D11 | Are you happy that your parents are on international migration? (आमाबाबु विदेशमा भएकोमा के तपाईं खुसी हुनुहुन्छ?) | 1. Yes (खुसि छु) 2. No (खुसी छैन) | |  |

| E | Adolescent's living arrangements (किशोरकिशोरीको बसाईको अवस्था) | | |
| --- | --- | --- | --- |
| **S.N.**  (क्र.सं) | **Questions**  (प्रश्नहरू) | **Answers**  **(उत्तरहरु)** | Skip |
| E1 | Who do you live with?  तपाईं अहिले कोसँग बस्नुहुन्छ? | 1. Parents (आमाबाबुसंग) 2. Family members other than parents (आमाबाबुबाहेक परिवारका अन्यसदस्यहरूसंग) 3. Relatives (नातेदारहरूसंग) 4. School hostel (स्कूल छात्रावासमा 5. Others (अन्य खुलाउनुहोस्) ....................................... |  |
| E2 | How do you rate your current living conditions (physical aspects)? (तपाई अहिले बसोवास गरिरहेको ठाउँ भौतिक पूर्वाधारको हिसाबले तपाईलाई कस्तो लाग्छ ?) | 1. Very good (धेरै राम्रो छ) 2. Satisfactory (सन्तोषजनक छ) 3. Poor (नराम्रो) 4. Very poor (धेरै नराम्रो) |  |
| E3 | Who is your primary caretaker? (अहिले बसिरहेको ठाउँमा तपाईको हेरविचार / स्याहारसुसार कसले बढि गर्छ ?) | 1. Mother (आमा) 2. Father (बुवा) 3. Grand Parents (हजुरबुवा /हजुरआमा) 4. Aunt (काकी- ठूलीआमा/सानीआमा/अन्टी) 5. Uncle (काका-ठूलोबुवा/सानोबुवा/अंकल) 6. Father’s Sister (फुपू/ फुपाजु) 7. Others अन्य(खुलाउनुहोस्)   ........................................ |  |
| E4 | How is your relationship with your primary caretaker? (तपाईको मुख्यरुपमा हेरविचार /स्याहारसुसार गर्ने व्यक्तिसँगको तपाईकोसम्बन्ध कस्तो छ ? | 1. Very good (धेरै राम्रो छ) 2. Satisfactory (सन्तोषजनक छ) 3. Poor (नराम्रो) 4. Very poor (धेरै नराम्रो) |  |
| E5 | Do you think your primary caretaker is caring and supportive to you? (के तपाईलाई मुख्यरुपमा हेरविचार/ स्याहारसुसार गर्ने व्यक्ति तपाईप्रति मायालु र सहयोगी हुनुहुन्छ जस्तो लाग्छ ?) | 1. Yes (मायालु र सहयोगी हुनुहुन्छ) 2. No (मायालु र सहयोगी हुनुहुन्न) |  |
| E6 | Do you have your own brothers or sisters living with you? (तपाई अहिले बसोवास गरिरहेको ठाउँमा के तपाईका आफ्नै दाजुभाई वा दिदीबहिनी सँगै बस्नुहुन्छ) | 1. Yes (सँगै बस्नुहुन्छ) 2. No (सँगै बस्नुहुन्न) 3. Don’t have brother sisters (दाजुभाई दिदीबहिनी हुनुहुन्न) | Go to **F1** |
| E7 | If you have your own brothers or sisters living with you, do you think he/she is caring and supportive to you? (यदि तपाईका आफ्नै दाजुभाई वा दिदीबहिनी सँगै बस्नुहुन्छ भने के उहाँहरु तपाईप्रति मायालु र सहयोगी हुनुहुन्छ?) | 1. Yes (मायालु र सहयोगी हुनुहुन्छ) 2. No (मायालु र सहयोगी हुनुहुन्न) |  |
| E8 | How many siblings do you have? (तपाईंका कति दाजुभाई दिदीबहिनी हुनुहन्छ?) | ………………. |  |

| **F.** | **Strength and Difficulty Questionnaire (**शक्ति तथा कठिनाइहरुको प्रश्नावली) | | | |
| --- | --- | --- | --- | --- |
|  | Please mark the box for Not True, Somewhat True, or Certainly True for each item. It would help us if you answered all items as best you can even if you are not certain or the item seems daft! Please answer based on how things have been for you over the last six months. (तलको तालिकाको हरेक विवरणका लागि कृपया बेठीक, केही मात्रामा ठीक वा निश्चयनै ठीक मध्ये एकलाई छानेर उपयुक्त कोठामा ठीक चिन्ह (√) लगाउनुहोस्। तपाई पूर्णरुपमा निश्चित हनुहुन्न भने पनि वा प्रश्न वाहियात लागेमा पनि तपाईले हरेक विवरणको जतिसक्दो उत्तम उत्तर दिनुभयो भने हामीलाई मद्दत मिल्नेछ । कृपया गत ६ महिनाभित्रको तपाईको अनुभवको आधारमा आफ्नो उत्तर दिनुहोस्।) | | | |
| **S.N.**  क्र. स | **Statements**  **बयानहरू** | **Not True**  (बेठीक) | **Somewhat True (केही मात्रामा ठीक)** | **Certainly True (नश्चयनै ठीक)** |
| F1 | I try to be nice to other people. I care about their feelings. (म अरूसँग राम्रो व्यवहार गर्ने कोसिस गर्छु। म तिनीहरूको भावनाको ख्याल राख्छु) | □ | □ | □ |
| F2 | I am restless, I find it hard to sit down for long (म बेचैन छु, म लामो समयसम्म स्थिर रहन सक्दिन) | □ | □ | □ |
| F3 | I get a lot of headaches, stomach-aches or sickness (मलाई धेरै टाउको दुख्छ, पेट दुख्छ, वा बिरामी हुन्छु) | □ | □ | □ |
| F4 | I usually share with others, for example, food or drink (मैले प्रायजशो खानेकुरा, खेलौना आदि अरु सँग बाँड्ने गर्छु | □ | □ | □ |
| F5 | I get very angry and often lose my temper (म धेरै रिसाउँछु, र प्रायजशो रिसमा नियन्त्रण गुमाउँछु) | □ | □ | □ |
| F6 | I am usually on my own. I generally play alone or keep to myself (म प्रायजशो आफ्नै धुनमा हुन्छु, म साधरणतयाः एक्लै खेल्छु वा आफ्नै शुरमा रहन रुचाउँछु) | □ | □ | □ |
| F7 | I am generally willing to do what other people want (म सामान्यतः मलाई जे गर्न भनिएको हुन्छ, त्यो गर्छु) | □ | □ | □ |
| F8 | I worry a lot (म धेरै चिन्ता गर्छु) | □ | □ | □ |
| F9 | I am helpful if someone is hurt, upset or feeling ill (यदि कोही घाइते, दुःखीत वा बिरामी छ भने म सहयोगी हुन्छु) | □ | □ | □ |
| F10 | I am constantly fidgeting or squirming (म निरन्तर छटपटाइरहन्छु वा मडारिईरहन्छु) | □ | □ | □ |
| F11 | I have one good friend or more (मेरा एक वा धेरै जना असल साथी छन्) | □ | □ | □ |
| F12 | I fight a lot. I can make other people do what I want (म धेरै पिटापिट गर्छु । म अन्य मानिसहरूलाई आँफूलाई जे चाह्यो त्यहि गराउन सक्छु) | □ | □ | □ |
| F13 | I am often unhappy, depressed or tearful (म प्रायजशो दुखि, निराश वा आँसुले टिलपिल हुन्छु) | □ | □ | □ |
| F14 | Other people generally like me (सामान्यतयाः मेरो उमेरका मानिसहरूले मलाई मनपराउँछन्) | □ | □ | □ |
| F15 | I am easily distracted; I find it difficult to concentrate (म सजिलै विचलित हुन्छु; मलाई ध्यान केन्द्रित गर्न गाह्रो हुन्छ) | □ | □ | □ |
| F16 | I am nervous in new situations. I easily lose confidence (म नयाँ परिस्थितिमा अतालिंछु। म सजिलै आत्मविश्वास गुमाउँछु) | □ | □ | □ |
| F17 | I am kind to younger children (म आँफूभन्दा साना बालबालिका प्रति दयालु छु) | □ | □ | □ |
| F18 | I am often accused of lying or cheating (मलाई बारम्बार झुठो बोलेको वा ठगेको दोष लाग्छ) | □ | □ | □ |
| F19 | Other children or young people pick on me or bully me (अन्य बालबालिका वा किशोरकिशोरीहरुले मलाई गिज्याउने वा धप्काउने तर्साउने गर्छन्) | □ | □ | □ |
| F20 | I often offer to help others (family members, friends, colleagues) {म प्रायजशो अरुको सहायताको लागि स्वयंसेवा गर्छु (बाबुआमा, शिक्षक, अरु बालबालिकाहरू)} | □ | □ | □ |
| F21 | I think before I do things (म केही गर्नुभन्दा पहिला सोच्छु) | □ | □ | □ |
| F22 | I take things that are not mine from home, school or elsewhere (म घर, विद्यालय वा अन्यत्रबाट मेरो स्वमित्वमा नभएका सामानहरु लैजान्छु) | □ | □ | □ |
| F23 | I get on better with adults than with people my own age (म मेरो उमेरका मानिसहरूसँग भन्दा वयस्कसँग बढी मिल्छु) | □ | □ | □ |
| F24 | I have many fears, I am easily scared (म धेरै डराउँछु, म सजिलै तर्सिन्छु) | □ | □ | □ |
| F25 | I finish the work I'm doing. My attention is good (म आफुले गरिरहेको काम पूरा गर्छु, मेरो ध्यान राम्रो छ) | □ | □ | □ |

Thank you for your participation in this study
