# Supplementary material for: Psychological well-being of adolescents in Pokhara, Nepal: A comparison between migrated and non-migrated parents
Source: PLOS Ment Health. 2025 Aug 12;2(8):e0000102. doi: 10.1371/journal.pmen.0000102 (PMC12798523; doi:10.1371/journal.pmen.0000102)
Supplement: S3 Data — (PDF) [file pmen.0000102.s003.pdf]

Strengths and Difficulties Questionnaire

For each item, please mark the box for Not True, Somewhat True or Certainly True. It would help us if you answered all items as best you can even if you are not absolutely certain or the item seems daft! Please give your answers on the basis of how things have been for you over the last six months.

Your Name .... Male/Female

Date of Birth..

	Not True	Somewhat True	Certainly True
I try to be nice to other people. I care about their feelings	☐	☐	☐
I am restless, I cannot stay still for long	☐	☐	☐
I get a lot of headaches, stomach-aches or sickness	☐	☐	☐
I usually share with others (food, games, pens etc.)	☐	☐	☐
I get very angry and often lose my temper	☐	☐	☐
I am usually on my own. I generally play alone or keep to myself	☐	☐	☐
I usually do as I am told	☐	☐	☐
I worry a lot	☐	☐	☐
I am helpful if someone is hurt, upset or feeling ill	☐	☐	☐
I am constantly fidgeting or squirming	☐	☐	☐
I have one good friend or more	☐	☐	☐
I fight a lot. I can make other people do what I want	☐	☐	☐
I am often unhappy, down-hearted or tearful	☐	☐	☐
Other people my age generally like me	☐	☐	☐
I am easily distracted, I find it difficult to concentrate	☐	☐	☐
I am nervous in new situations. I easily lose confidence	☐	☐	☐
I am kind to younger children	☐	☐	☐
I am often accused of lying or cheating	☐	☐	☐
Other children or young people pick on me or bully me	☐	☐	☐
I often volunteer to help others (parents, teachers, children)	☐	☐	☐
I think before I do things	☐	☐	☐
I take things that are not mine from home, school or elsewhere	☐	☐	☐
I get on better with adults than with people my own age	☐	☐	☐
I have many fears, I am easily scared	☐	☐	☐
I finish the work I'm doing. My attention is good	☐	☐	☐

Your signature .... Today's date ....

Thank you very much for your help

© Robert Goodman, 2005
