# Supplementary material for: Psychological well-being of adolescents in Pokhara, Nepal: A comparison between migrated and non-migrated parents
Source: PLOS Ment Health. 2025 Aug 12;2(8):e0000102. doi: 10.1371/journal.pmen.0000102 (PMC12798523; doi:10.1371/journal.pmen.0000102)
Supplement: S1 Table — (DOCX) [file pmen.0000102.s005.docx]

S1 Table: Socio-demographic characteristics of adolescents

| **Variables** | **Overall** | | **Adolescents of Migrated Parents** | | **Adolescents of Non-Migrated Parents** | |
| --- | --- | --- | --- | --- | --- | --- |
|  | n | % | n | % | n | % |
| **Age** |  |  |  |  |  |  |
| Early adolescents (12-14) | 425 | 58.70 | 206 | 56.90 | 219 | 60.50 |
| Late adolescents (15-17) | 299 | 41.30 | 156 | 43.10 | 143 | 39.50 |
| Age (Years) Mean (S.D.) 14.33 (.969) | | |  |  |  |  |
| **Sex** |  |  |  |  |  |  |
| Female | 363 | 50.10 | 184 | 50.80 | 179 | 49.40 |
| Male | 361 | 49.90 | 178 | 49.20 | 183 | 50.60 |
| **Ethnicity** |  |  |  |  |  |  |
| Janajati | 258 | 35.60 | 139 | 38.40 | 119 | 32.90 |
| Brahmin | 228 | 31.50 | 85 | 23.50 | 143 | 39.50 |
| Chhetri | 135 | 18.60 | 70 | 19.30 | 65 | 18 |
| Dalit | 90 | 12.40 | 65 | 18 | 25 | 6.90 |
| Madhesi | 8 | 1.10 | 1 | 0.30 | 7 | 1.90 |
| Muslim | 5 | 0.70 | 2 | 0.60 | 3 | 0.80 |
| **Religion** |  |  |  |  |  |  |
| Hinduism | 603 | 83.30 | 293 | 80.90 | 310 | 85.60 |
| Buddhism | 88 | 12.20 | 50 | 13.80 | 38 | 10.50 |
| Christianity | 27 | 3.70 | 16 | 4.40 | 11 | 3 |
| Islam | 6 | 0.80 | 3 | 0.80 | 3 | 0.80 |
| **Parental education** |  |  |  |  |  |  |
| Illiterate | 3 | 0.40 | 0 | 0 | 3 | 0.80 |
| Can read and write only | 34 | 4.70 | 12 | 3.30 | 22 | 6.10 |
| Basic school | 120 | 16.60 | 71 | 19.60 | 49 | 13.50 |
| Secondary school | 376 | 51.90 | 209 | 57.70 | 167 | 46.10 |
| Bachelor’s and above | 191 | 26.40 | 70 | 19.30 | 121 | 33.40 |
| **Socio – economic status** |  |  |  |  |  |  |
| Very low | 152 | 21 | 72 | 19.90 | 80 | 22.10 |
| Low | 140 | 19.30 | 74 | 20.40 | 66 | 18.20 |
| Middle | 151 | 20.90 | 75 | 20.70 | 76 | 21 |
| High | 196 | 27.10 | 117 | 32.30 | 79 | 21.80 |
| Very high | 85 | 11.70 | 24 | 6.60 | 61 | 16.90 |
| **Type of family** |  |  |  |  |  |  |
| Nuclear family | 449 | 62 | 229 | 63.30 | 220 | 60.80 |
| Joint family | 239 | 33 | 116 | 32 | 123 | 34 |
| Extended family | 36 | 5 | 17 | 4.70 | 19 | 5.20 |
| **Type of schools** |  |  |  |  |  |  |
| Private School | 389 | 53.70 | 197 | 54.40 | 192 | 53 |
| Government School | 335 | 46.30 | 165 | 45.60 | 170 | 47 |
